# ﻿Comparative mitogenomics, phylogeny, and biogeography of selected species of *Saxicola* (Aves, Passeriformes)

**DOI:** 10.3897/zookeys.1249.152269

**Published:** 2025-08-13

**Authors:** Chuan Jiang, Wenwen Zhu, Shujia Wu, Xilong Zhao, Nassoro Mohamed, Bo Li

**Affiliations:** 1 College of Wildlife and Protected Area, Northeast Forestry University, Harbin 150040, China Northeast Forestry University Harbin China; 2 School of Ecology, Sun Yat-Sen University, Shenzhen 518107, China Sun Yat-Sen University Shenzhen China; 3 College of Life Science and Technology, Jinan University, Guangzhou 510632, China Jinan University Guangzhou China; 4 Shenzhen Frontier Science and Innovation Research Center, Shenzhen 518000, China Shenzhen Frontier Science and Innovation Research Center Shenzhen China; 5 College of African Wildlife Management, Mweka, P.O Box 3031, Moshi, Kilimanjaro, Tanzania College of African Wildlife Management Moshi Tanzania; 6 State Forestry and Grassland Administration Detecting Center of Wildlife, Harbin 150040, China State Forestry and Grassland Administration Detecting Center of Wildlife Harbin China

**Keywords:** Classification, divergence time, mitochondrial genome, stonechat

## Abstract

*Saxicola* is a genus of insectivorous birds widely distributed across Eurasia and Africa. Their mitochondrial genomes (mitogenomes), classification, phylogeny, and biogeography are underresearched. Here, we sequenced and/or assembled the mitogenomes of *S.stejnegeri*, *S.torquatus*, *S.maurus*, *S.rubicolahibernans*, *S.r.rubicola* and *S.dacotiae* for detailed comparative analysis, integrating them with published mitogenomes of members of Muscicapidae for phylogenetic reconstruction. Additionally, we estimated the divergence times and ancestral ranges for *Saxicola* using *ND2* and *Cytb*. These mitogenomes, spanning 16,764 bp to 16,804 bp, exhibit typical gene arrangement, AT bias, and A/C skew tendencies. Among the 13 protein-coding genes, *ND3* evolved the fastest, whereas *COX1* evolved the slowest. The 22 *tRNAs* form the classic cloverleaf structure except *trnS1* (AGY). The control region has three domains with 10 conserved elements. Mitogenomic phylogenetic analysis revealed the topology: (((((*S.r.hibernans* + *S.r.rubicola*) + *S.dacotiae*) + *S.maurus*) + *S.torquatus*) + *S.stejnegeri*), supporting the independent species status of *S.stejnegeri* rather than a subspecies of *S.maurus*. Ancestral range reconstruction indicated that *Saxicola* originated in the Indian subcontinent and mainland Southeast Asia during the late Miocene. These findings enhance our understanding of the classification, evolution, and geographical origin of *Saxicola*.

## ﻿Introduction

*Saxicola* (stonechats) is a genus of small passerine birds belonging to the family Muscicapidae. The genus consists of widely distributed insectivorous bird species that are found across Asia, Europe, and Africa, inhabiting open habitats with woody shrubs, ranging from coastal areas to mountainous alpine environments (Winkler et al. 2020). The taxonomy of this genus remains contentious, with various checklists recognizing 13–15 species (Clements et al. 2024; Gill et al. 2024; AviList Core Team 2025), most notably the widely distributed *S.torquatus* complex across Palaearctic and Afrotropical regions (Illera et al. 2008; Zink et al. 2009; Svensson et al. 2012; Opaev et al. 2018). Based on distribution, morphology, vocalizations, habitat preference, mitochondrial genes and genomic data (Wittmann et al. 1995; Urquhart 2002; Illera et al. 2008; Zink et al. 2009; Svensson et al. 2012; Hellström and Norevik 2014; Van Doren et al. 2017; Opaev et al. 2018; Loskot and Bakhtadze 2020; Hellström and Waern 2023), the *S.torquatus* complex is currently classified into 5–7 species: *S.stejnegeri* (or *S.maurusstejnegeri*), *S.torquatus*, *S.sibilla* (or *S.torquatussibilla*), *S.tectes*, *S.maurus*, *S.dacotiae*, and *S.rubicola* (Clements et al. 2024; Gill et al. 2024; AviList Core Team 2025). Among them, the recognition of *S.stejnegeri* as a distinct species primarily stems from its phylogenetically distinct from the taxon *S.maurus* based on a single mitochondrial gene (Zink et al. 2009), though its relationship with *przewalskii* and *indicus* (taxa with nomenclatural priority) remains unresolved (Sangster et al. 2011; Chesser et al. 2022). Nevertheless, due to its parapatric distribution and potential hybridization with *S.maurus* (Hellström and Norevik 2014; Hellström and Waern 2023), some scholars still consider it to be a subspecies of *S.maurus* (AviList Core Team 2025). This ambiguity is also partly attributed to previous studies on this species complex relying on single mitochondrial genes, which have not yielded a conclusive phylogeny, emphasizing the need for high-resolution molecular markers to untangle this species complex.

Ancestral area reconstruction is one of the primary goals of biogeography research, which aims to investigate the geographical origins, dispersal, and evolutionary history of taxa, thereby understanding their current distribution patterns and environmental adaptations (Dupin et al. 2017). Illera et al. (2008) studied the biogeography of *Saxicola* based on *Cytb* through DIVA analysis, proposing that this genus originated in Asia, with subsequent dispersal and diversification in four main directions, eventually occupying Eurasia and Africa. However, owing to the limited phylogenetic information provided by the single *Cytb* and incomplete sampling (notably lacking samples from the eastern Palearctic), this study can serve only as a preliminary framework for the dispersal and diversification history of the genus. With continuous taxonomic revisions, the accumulation of more available gene data, and developments in statistical methods, this framework will continue to evolve and improve.

The mitochondrial (mt) genome (mitogenome), characterized by maternal inheritance in most taxa (such as birds), rapid coalescence rates, rare recombination, and suitable gene content, serves as a cornerstone for phylogenetic, taxonomic, and biogeographic studies in birds and other organisms (Vázquez-López et al. 2024). It is particularly valuable for inferring evolutionary events from the past 20 million years (Lavrov and Pett 2016). Additionally, features of mitogenomes, including nucleotide composition, codon usage, the secondary structures of RNAs, and the composition of control region (*CR*), also contain critical information about species differentiation. By comparing these characteristics among different taxa, unique and detailed insights into the evolutionary history of species can be provided.

The development of sequencing technologies has made mitogenomes available for an increasing number of avian taxa; however, this excellent marker remains poorly documented in *Saxicola*. In this study, we sequenced and/or assembled the mitogenomes of *S.stejnegeri*, *S.torquatus*, *S.maurus*, *S.dacotiae*, *S.r.hibernans*, *S.r.rubicola*, and conducted comparative analyses to characterize their features and reveal key information on species differentiation. These data were subsequently combined with 39 mitogenomes downloaded from NCBI for phylogenetic analysis to investigate the phylogenetic relationships inter- and intra-generic *Saxicola*, providing guidance for the classification of *Saxicola* species. Finally, utilizing concatenated sequences of the *Cytb* and *ND2* genes from 14 phylogenetic species of *Saxicola*, we conducted ancestral range reconstruction to refine the origin of this genus.

## ﻿Materials and methods

### ﻿Sample collection, sequencing, and data preparation

A bird carcass, seized by the State Forestry and Grassland Administration Detecting Center of Wildlife (Harbin, China) in Beian, Heilongjiang, was identified as *S.stejnegeri* based on its location and dark plumage. Due to legal restrictions on retaining seized wildlife materials, the specimen could not be deposited in a museum, but photographs documenting key diagnostic features are publicly available (https://www.inaturalist.org/observations/292680831). Total genomic DNA was extracted from muscle tissue of this carcass via a tissue extraction kit (UElandy, Suzhou, China). The high-quality extracted DNA was first fragmented by ultrasonic shearing, and then a library with an insert size of ~350 bp was constructed on the DNBSEQ platform following the manufacturer’s instructions (MGIEasy Universal DNA Library Preparation Kit, BGI). The libraries were sequenced on the DNBSEQ-T1 sequencer (MGI, China) with 150 bp paired-end strategy.

Furthermore, we downloaded the raw genome sequence fragment data for *S.maurus* (SRR17072840) (Bergeron et al. 2023), *S.r.rubicola* (ERS1563315), *S.r.hibernans* (ERS1563316), *S.torquatus* (ERS1563317) and *S.dacotiae* (ERS1563318) (Van Doren et al. 2017) from the SRA database for mitogenome assembly.

### ﻿Mitogenome assembly, annotation, and comparative analysis

All data were first filtered using fastp v. 0.23.4 (Chen 2023) with default parameters. Subsequently, the mitogenome sequences of six *Saxicola* taxa were *de novo* assembled from clean reads using GetOrganelle v. 1.7.5 (Jin et al. 2020). NOVOPlasty v. 4.3.5 (Dierckxsens et al. 2017) was also used for assembly to verify the structural correctness. Subsequently, we mapped the clean reads to the assembled mitogenome using BWA v. 0.7.18 (Jung and Han 2022) and calculated the coverage depth using Samtools v. 1.21 (Danecek et al. 2021). Mt genes were annotated using MITOS v. 2.1.9 (Zea et al. 2017). The annotation of *tRNAs* referred to the results of tRNAscan-SE v. 1.21 (Lowe and Chan 2016). Annotation of all mt genes was checked by aligning them with those of other Passeriformes species from GenBank.

The mitogenome maps were rendered in GeSeq v. 1.43 (Tillich et al. 2017). Nucleotide composition was calculated and analyzed using Geneious v. 10.1.3 (Kearse et al. 2012). Composition skew values were determined using the formulas AT-skew = [A − T]/[A + T] and GC-skew = [G − C]/[G + C] (Perna and Kocher 1995). Nonsynonymous substitution rates (Ka) and synonymous substitution rates (Ks) were calculated using DnaSP v. 6.0.7 (Rozas et al. 2017). Nucleotide diversity (*Pi*) was calculated using DnaSP v. 6.0.7 (Rozas et al. 2017) with a window size of 100 bp and a step size of 10 bp. Relative synonymous codon usage (RSCU) and amino acid count were computed using Phylosuit v. 1.2.3 (Xiang et al. 2023). RNA secondary structures were predicted using MITOS v. 2.1.9 (Zea et al. 2017). Conserved elements in the *CRs* were analyzed with reference to previous studies (Saccone et al. 1991; Delport et al. 2002; Bi et al. 2019; Jing et al. 2020; Jiang et al. 2024). The variable sites in the *CRs* were calculated using the R package seqinr (Gouy et al. 1984).

### ﻿Mitogenomic phylogenetic analyses

To determine the phylogenetic position of the genus *Saxicola* within Muscicapidae family and enhance the robustness of the phylogeny within *Saxicola*, we retrieved the mitogenomes of 38 Muscicapidae species from GenBank (www.ncbi.nlm.nih.gov/Genbank), along with the mitogenome of a Turdidae species, *Turduseunomus*, as an outgroup (Suppl. material 1: table S1). Notably, previous studies identified mitogenome misannotations in two Muscicapidae species on NCBI: *Cyanoptilacyanomelana* (NC_015232) is actually *Cyornishainanus* or *C.rubeculoides*, and *Muscicapagriseisticta* (NC_045181) is *M.sibirica* (Sangster and Luksenburg 2021). Phylogenetic analysis was conducted on 13 protein-coding genes (PCGs) using both maximum likelihood (ML) and Bayesian inference (BI) methods. These PCGs were initially aligned using MAFFT v. 7.313 (Katoh and Standley 2013) and subsequently refined using MACSE v. 2.06 (Ranwez et al. 2018). The alignment sites suitable for phylogenetic analysis were selected using Gblocks v. 0.91b (Talavera and Castresana 2007) and then concatenated using the ‘concatenate sequence’ plugin in PhyloSuite v. 1.2.3 (Xiang et al. 2023). The optimal partitioning scheme for nucleotide substitution models was determined by PartitionFinder v. 2.0 (Lanfear et al. 2017) based on the AICc criterion (Suppl. material 2: table S2). ML analyses were performed using IQ-TREE v. 1.6.8 (Nguyen et al. 2015) with 1,000 bootstraps. BI analyses were carried out using MrBayes v. 3.2 (Ronquist et al. 2012), employing 10,000,000 generations and 4 chains, with sampling conducted every 1,000 generations. The convergence and mixing of the chains were assessed using Tracer v. 1.6.1 (Rambaut et al. 2018) to ensure that all ESS values were above 200. The consensus tree was generated after discarding the initial 25% of the trees as burn-in.

### ﻿Divergence and biogeographic analyses of *Saxicola*

To estimate the divergence time of *Saxicola* taxa, we retrieved available *ND2* and *Cytb* sequences of this genus and *Oenanthemelanoleuca* (as outgroup) from NCBI (Suppl. material 3: table S3). After obtaining the alignment and partition model using the same steps as the mitogenome phylogenetic analysis, the divergence time was estimated using BI in BEAST v. 1.8.4 (Drummond and Rambaut 2007). A Yule model was selected as the tree prior. A relaxed uncorrelated lognormal distribution was used as a clock model for all genes; clock rates (ucld.mean parameters) were estimated by implementing a prior from a published substitution rate (Weir and Schluter 2008) of 0.0105 substitutions/site/million year for *Cytb*, while a uniform prior was used for *ND2*. The secondary calibration points were selected from the crown age of *Saxicola* genus 7.6 million years ago (mya) (Zhao et al. 2023) and 4.58 mya for *S.ferreus* and *S.jerdoni* from TimeTree (Kumar et al. 2022). MCMC analysis was performed twice for 50 million generations, with sampling every 1,000 generations. The convergence of each run was checked based on whether all parameters had an effective sample size greater than 200, and the first 25% of the trees were discarded as burn-in using Tracer v. 1.6.1 (Rambaut et al. 2018). The two independent runs were then combined using LogCombiner v. 2.6.4 (Bouckaert et al. 2014), and the resulting trees were summarized using TreeAnnotator v. 2.6.4 (Bouckaert et al. 2014) to generate the maximum clade credibility tree. The outgroup of the time tree was pruned using the R package ape (Paradis and Schliep 2019).

Based on the time-calibrated ultrametric tree obtained above, we used BioGeoBEARS (Matzke 2018) in R to reconstruct the most probable ancestral area for *Saxicola*. BioGeoBEARS enables the fitting of probabilistic biogeographic models using user-defined geographical areas. We defined eight geographical areas in the Old World based on the zoogeographical realms estimated in Holt et al. (2013) with adaptations: A) Indian subcontinent plus mainland Southeast Asia, B) Central Asian arid, C) Eastern Asia, D) Northeast Palearctic, E) Malay Archipelago, F) Western Palearctic, G) Afrotropical, and H) Réunion. Each stonechat taxon was coded as either present or absent in the designated geographical areas, following the breeding range information obtained from BirdLife International (Winkler et al. 2020). We tested six biogeographic models, including DEC, DIVA, and BayArea, along with their respective extensions (DEC+J, DIVALIKE + J, BayArea + J) that account for founder event speciation. The DEC, DIVA, and BayArea models all account for anagenetic processes, including dispersal and extinction, but they differ in their assumptions about cladogenetic processes (sympatry and vicariance). DEC requires that one daughter lineage is always assumed to have a range of only one area (Ree and Smith 2008). DIVA does not have this requirement but requires that daughter lineages occupy completely overlapping ranges under sympatric cladogenesis (Ronquist 1997; Yu et al. 2010). In contrast, BayArea requires that daughter lineages must inherit a range identical to their ancestor (Landis et al. 2013). The extended versions of these models incorporate a ‘J’ parameter, allowing one daughter lineage to inherit the ancestral range while the other disperses to occupy a new area beyond the ancestral range (Matzke 2014).

The fitting performance of the six models was evaluated through the weighted AICc (Suppl. material 4: table S4). The maximum range size was set as three since no taxa in the dataset had breeding ranges spanning more than three realms. Time-stratified analyses were employed to ensure that Réunion was not considered as the ancestral area for any species before its emergence 2.1 mya (Warren et al. 2003). Dispersal multipliers were utilized to regulate dispersal between areas, with a dispersal multiplier value of 1 for adjacent areas and 0.1 or 0.01 for nonadjacent areas (depending on the number of intervening areas) in accordance with the suggestions of Dupin et al (Dupin et al. 2017).

## ﻿Result and discussion

### ﻿Genome organization and composition

**Figure 1.**
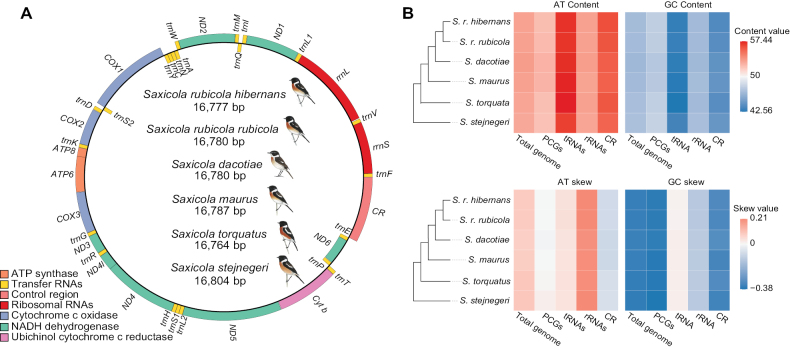
Mitogenome structure and nucleotide features. A. Mitogenome maps of six *Saxicola* taxa revealed a conserved ancestral gene order (Gibb et al. 2007). The genes on the H strand are outer, and the L strand genes are inner. The bird images were sourced from Birds of the World (2025); B. The heatmap of nucleotide features revealed interspecies similarities and differences among gene types.

The avian mitogenome typically ranged from 16 to 17 kp, but it could be expanded due to rearrangements, pseudogenes, or tandem repeats in the *CR*, as observed in *Penelopidespanini* with a length of 22,737 bp (Sammler et al. 2011). All six mitogenomes were annotated with 37 genes each (13 PCGs, 22 *tRNAs*, and 2 *rRNAs*), with no pseudogenes or tandem repeats detected. Structural integrity was confirmed by coverage depth plots (Suppl. material 5: fig. S1), though a decrease in depth was observed in *ND6* at around 15,000 bp in each species, potentially due to sequencing biases or high heteroplasmy in that gene. Mitochondrial gene orders were conserved among species, following the *Cytb*-*trnT*-*trnP*-*ND6*-*trnE*-*CR* arrangement (Fig. 1a, Suppl. material 6: table S5), akin to the ancestral gene order in chicken (*Gallusgallus*) (Gibb et al. 2007), with lengths ranging from 16,764 bp to 16,804 bp, which fell within the normal range (Boore 1999). Notably, the mitogenomes of *S.r.hibernans*, *S.r.rubicola*, *S.dacotiae*, and *S.torquatus* were assembled from pooled sequencing; they represented pseudo-haplotypes of these species.

The nucleotide compositions and skewness were generally similar among the mitogenomes of six species of *Saxicola*, but differences were observed between the total genome and different gene types (Fig. 1b, Suppl. material 7: table S6). The A+T content was higher than the G+C content in both the total genome and all gene types of the six mitogenomes, indicating an A+T bias, which was most significant in *tRNAs* and the *CR*. The AT skews of the six mitogenomes were all positive (0.0777~0.0942), while the GC skews were negative (-0.3627~-0.3509), indicating strand asymmetry with more As than Ts and more Cs than Gs. For different gene types, significant A skew was observed in *rRNAs*, moderate A skew and T skew were observed in *tRNAs* and *CR*, respectively, and slight A skew (in *S.stejnegeri*, *S.torquatus* and *S.r.hibernans*) or T skew (in *S.maurus*, *S.dacotiae* and *S.r.rubicola*) was observed in PCGs. The C skew was significant in the PCGs and *CR*, moderate in *rRNAs*, and a slight G skew was observed in *tRNAs*. These features were also observed in the mitogenomes of other species of Muscicapidae (Lan et al. 2024; Yuan et al. 2024) and passerine birds in general (Yang et al. 2022; Jiang et al. 2024).

### ﻿Protein-coding genes

Animal mitogenomes typically contained 13 PCGs, with *ND6* encoding on the L-strand and the remaining 12 encoding on the H-strand (Boore 1999), a pattern also observed in the mitogenomes of the six species of *Saxicola* (Fig. 1a). The lengths of these 13 PCGs were conserved across the six *Saxicola* mitogenomes, ranging from 168bp for *ATP8* to 1,818 bp for *ND5*, with a total length of 11,369 bp (Suppl. material 6: table S5).

**Figure 2.**
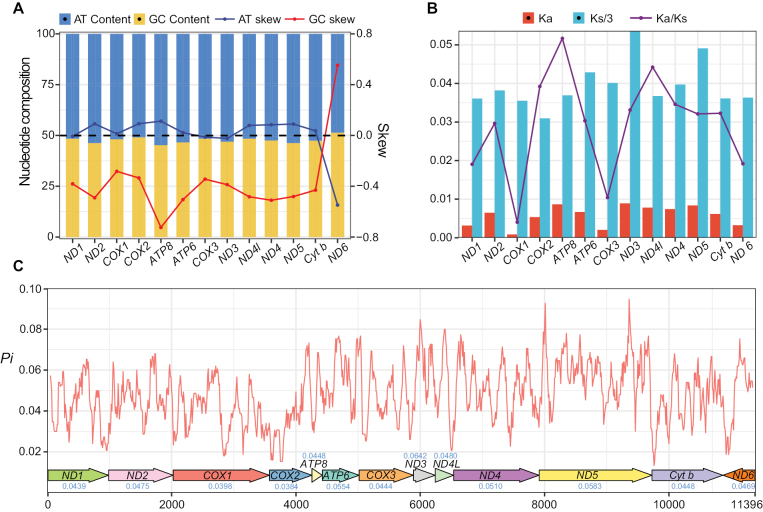
Nucleotide features, selection pressure, and nucleotide diversity of protein-coding genes (PCGs) in the six mitogenomes revealed inter-gene difference. A. The base composition and skewness of PCGs averaged across six mitogenomes; B. Ka, Ks, and Ka/Ks values of PCGs across 6 *Saxicola* taxa indicated that *ND3* evolved the fastest, while *COX1* evolved the slowest. Ka was divided by 3 (Ka/3) for better visualization; C. *Pi* value of the concatenated PCGs sliding window (window size = 100 bp, step size = 10 bp). The arrows below the line graph represent genes, with direction indicating gene orientation. The blue numbers above or below the arrows indicate the *Pi* for each gene.

To explore the evolutionary patterns of PCGs, we conducted analyses on nucleotide composition, skewness, selection pressure (by the Ka/Ks ratio), and *Pi* for each PCG (Fig. 2a). The nucleotide composition and skewness of PCGs were typically associated with gene function, expression levels, and DNA replication and repair mechanisms (Lercher et al. 2003; Merino et al. 2019). In our case, 13 PCGs exhibited similar AT contents, ranging from 48.61% for *ND6* to 54.76% for *ATP8*. In addition to *ND6*, which showed a G skew, the other 12 PCGs all displayed a C skew, with *ATP8* exhibiting the strongest C skew. Four PCGs (*ND1*, *ND3*, *ND6*, *COX3*) showed a T skew, most pronounced in *ND6*, while the remaining nine exhibited an A skew.

Ka and Ks represented the rates of nonsynonymous and synonymous mutations, respectively, with their Ka/Ks ratios serving as excellent indicators of selection pressure on PCGs. Ka/Ks = 1 indicated neutral selection, a value greater than 1 indicated diversifying selection, and a value less than 1 indicated purifying selection (Hurst 2002). The Ka/Ks ratios of all 13 PCGs were far lower than 1 (Fig. 2b), suggesting that their evolution was under purifying selection. In particular, *COX1* had minimal Ka and Ka/Ks ratios, highlighting its high conservation and pivotal role in mitogenome functionality.

Nucleotide diversity (*Pi*) referred to the average number of nucleotide differences per site between pairs of sequences, reflecting the degree of genetic variation and selection pressure at different sites (Nei and Li 1979; Wang et al. 2024). Sliding window analysis along the concatenated sequences of the 13 PCGs revealed wide variation in *Pi* per site, with notable decreases in the latter half of *COX1* and the front half of *COX2* (Fig. 2c). For each PCG, *Pi* was lowest in *COX1* (0.0398) and *COX2* (0.0384), while highest in *ND3* (0.0642). Combining the lowest Ka/Ks ratio of *COX1* and the highest Ka value of *ND3*, it could be concluded that *COX1* was the slowest evolving gene, while *ND3* was the fastest evolving gene. The slower evolution of *COX1* further supported its utility as a barcode for identifying species of Metazoa (Bianchi and Gonçalves 2021).

**Figure 3.**
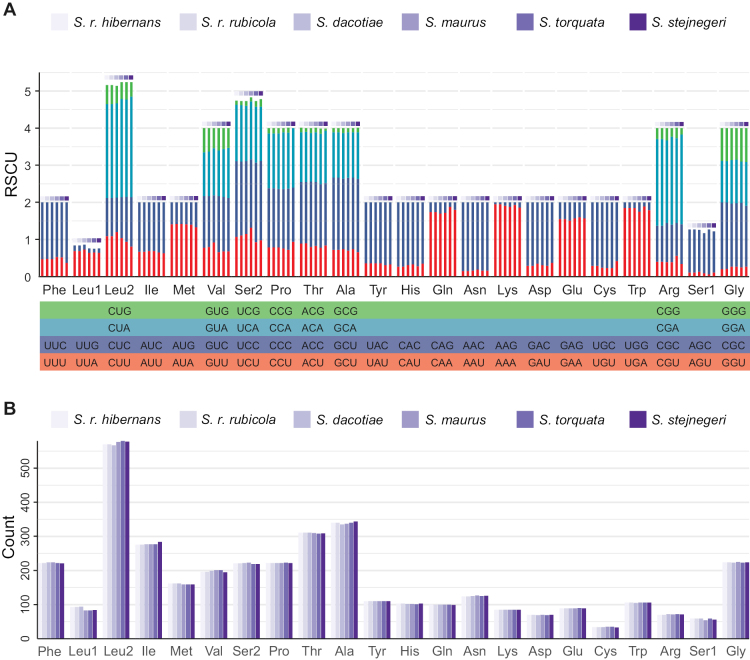
Codon and amino acid usage patterns in the six mitogenomes demonstrated evolutionary conservation across species. A. The RSCU values of the PCGs showed a preference for codons ending in A and C; B. Amino acid composition of the PCGs showed that amino acids with more synonymous codons were used more frequently.

We also compared the start and stop codons, RSCU, and amino acid usage of PCGs among six *Saxicola* mitogenomes. All PCGs started with ATG, while the termination codons included TAA, TAG, AGG, AGA, and T, which were conserved across species except for *ND6* (Table 1). The codon usage pattern and amino acid quantities were generally consistent among species but exhibited significant differences across different amino acids (Fig. 3), revealing the evolutionary selection pressure of different amino acids. Generally, amino acids with more synonymous codons tended to have higher usage frequencies (Bulmer 1991; Yang et al. 2024). In this study, amino acids with four synonymous codons mostly showed greater abundance compared to other amino acids, such as Leu2, Val, Ser 2, Pro, Thr, Ala, and Gly. Interestingly, despite their lower quantities, Phe and Ile showed higher abundance, suggesting potentially more stable genetic efficiency for these two amino acids (Ray et al. 2014). Furthermore, RSCU analysis indicated that codons ending with A and C were more frequent than those ending with U and G (Fig. 2a), consistent with the A skew and C skew observed in the mitogenome (Figs 1–3).

**Table 1.** Initial and terminal codons for protein-coding genes. Codons with interspecies variation are highlighted in bold.

Species	Gene												
	*ND1*	*ND2*	*COX1*	*COX2*	*ATP8*	*ATP6*	*COX3*	*ND3*	*ND4L*	*ND4*	*ND5*	*Cytb*	*ND6*
*Saxicolarubicolahibernans*	ATG/AGG	ATG/TAA	ATG/AGG	ATG/TAA	ATG/TAA	ATG/TAA	ATG/T	ATG/TAA	ATG/TAA	ATG/T	ATG/AGA	ATG/TAA	ATG/**TAG**
*Saxicolarubicolarubicola*	ATG/AGG	ATG/TAA	ATG/AGG	ATG/TAA	ATG/TAA	ATG/TAA	ATG/T	ATG/TAA	ATG/TAA	ATG/T	ATG/AGA	ATG/TAA	ATG/**TAG**
*Saxicoladacotiae*	ATG/AGG	ATG/TAA	ATG/AGG	ATG/TAA	ATG/TAA	ATG/TAA	ATG/T	ATG/TAA	ATG/TAA	ATG/T	ATG/AGA	ATG/TAA	ATG/**TAG**
*Saxicolamaurus*	ATG/AGG	ATG/TAA	ATG/AGG	ATG/TAA	ATG/TAA	ATG/TAA	ATG/T	ATG/TAA	ATG/TAA	ATG/T	ATG/AGA	ATG/TAA	ATG/**TAA**
*Saxicolatorquatus*	ATG/AGG	ATG/TAA	ATG/AGG	ATG/TAA	ATG/TAA	ATG/TAA	ATG/T	ATG/TAA	ATG/TAA	ATG/T	ATG/AGA	ATG/TAA	ATG/**TAG**
*Saxicolastejnegeri*	ATG/AGG	ATG/TAA	ATG/AGG	ATG/TAA	ATG/TAA	ATG/TAA	ATG/T	ATG/TAA	ATG/TAA	ATG/T	ATG/AGA	ATG/TAA	ATG/**TAG**

### ﻿RNA genes

Similar to other avian mitogenomes (Yang et al. 2022), each of the mitogenomes of the six species of *Saxicola* contained 2 *rRNAs* and 22 *tRNAs*. Among these, eight tRNAs (*trnQ*, *trnA*, *trnN*, *trnC*, *trnY*, *trnS2*, *trnP*, and *trnE*) were encoded on the L-strand, while the rest were encoded on the H-strand (Fig. 1a).

In the six *Saxicola* mitogenomes, the length of *rrnL* ranged from 1581 to 1583, while the length of *rrnS* ranged from 982 to 984 (Suppl. material 6: table S5). Besides the varying length of *trnC*, the lengths of the *tRNAs* were conserved, ranging from 67 bp (*trnS1*) to 75 bp (*trnS2*).

**Figure 4.**
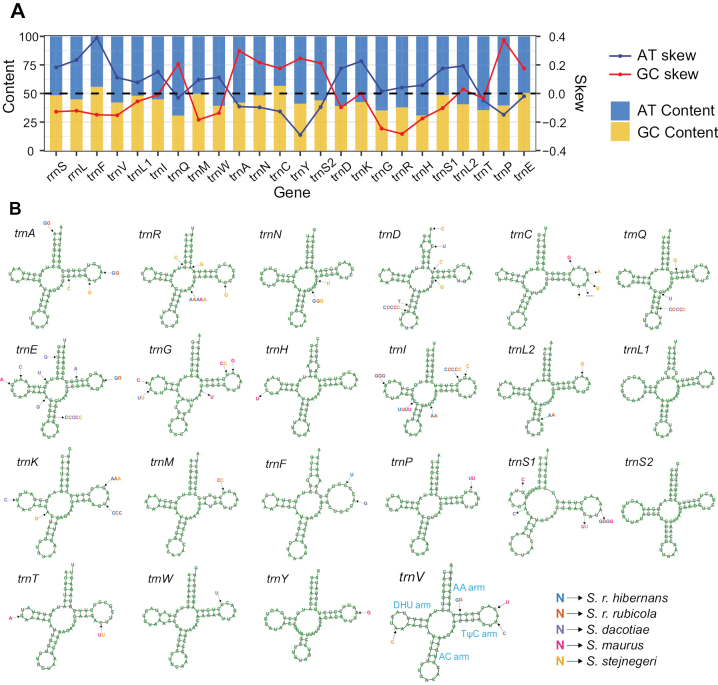
Nucleotide features of 22 *tRNAs* in six mitogenomes and their products’ secondary structures. A. The base composition and skewness of 22 *tRNAs* averaged across six mitogenomes revealed inter-gene difference; B. The secondary structures of 22 *tRNAs* of *S.torquatus* revealed a conserved cloverleaf structure. The black arrows indicated variable sites between taxa, with variable bases shown in different colored letters, and ‘-’ indicated base deletions.

To examine the differences among various RNA genes, we calculated the average nucleotide composition and skewness for each RNA gene across six *Saxicola* mitogenomes. The results revealed significant variations, with *trnC* showing the highest GC content (56.73%) and *trnQ* exhibiting the lowest (30.73%) (Fig. 4a). In terms of AT skew, 15 RNA genes presented an A skew, and nine presented a T skew. For the GC skew, 14 RNA genes presented a C skew, and ten presented a G skew. These inter-gene differences might be related to the functions of different RNAs and their different locations in mitogenome (Tillier and Collins 2000; Butenko et al. 2024).

Like other birds (Yang et al. 2022), except for the absence of the dihydrouracil arm in *trnS1*, all *tRNAs* in the mitogenomes of the six species of *Saxicola* formed a cloverleaf structure consisting of the amino acid acceptor (AA) arm, anticodon (AC) arm, dihydrouracil arm (DHU arm), and TyC (T) arm (Fig. 4b). A total of 65 variable sites were detected among the mitogenomes of the six species of *Saxicola*, appearing in 20 *tRNAs*. The most frequent appearance was *trnE*, with a total of eight variable sites identified.

### ﻿Control region

The *CR* was usually considered to be the most variable in the mitogenome, and consisted of three domains: extended termination-associated sequences (ETAS), central conserved domain (CCD), and conserved sequence block (CSB) (Saccone et al. 1991; Bi et al. 2019). This region regulated the transcription and replication of mitogenomes and contained some conserved motifs that might have function-associated secondary structures, such as CSB-1 (Delport et al. 2002). The *CRs* of the six *Saxicola* mitogenomes varied in length from 1,202 bp in *S.torquatus* to 1,244 bp in *S.stejnegeri* (Suppl. material 6: table S5). Length differences were primarily observed in the ETAS (407–409 bp) and CSB (363–405 bp), while the CCDs were conserved at 431 bp inter-specifically.

**Figure 5.**
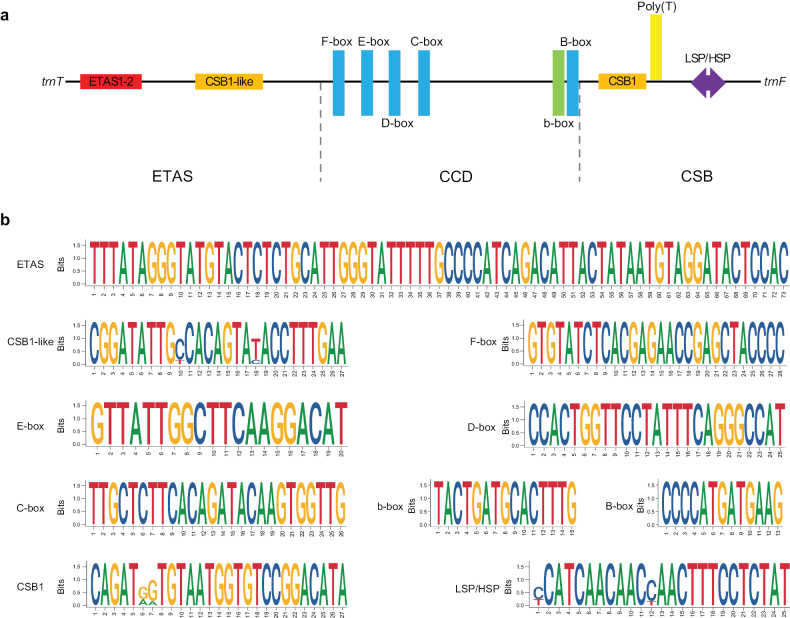
Structural features of the control region (*CR*) of six *Saxicola* mitogenomes. A. The structure of *CR* displayed three domains with 10 conserved elements. Different colors were used to represent distinct motifs. The size of the motifs was not scaled; B. The sequence logo of conserved elements in *CR* revealed variations in evolutionary rates among different elements.

As expected, ten conserved sequence elements were identified in the *CR*, including ETAS1-2 and CSB-like in ETAS, F, E, D, C, B boxes and Bird similarity box in CCD, as well as CSB-1 and LSP/HSP in CSB (Fig. 5a). These conserved sequence boxes were also identified in other avian mitogenomes (Bi et al. 2019; Jing et al. 2020).

The evolutionary rates of the three domains varied significantly, with percentages of variable nucleotides in the three domains being 10.51%, 3.38%, and 21.99%, respectively. Most of the variable sites were concentrated in domains 1 and 3, which was consistent with previous studies (Ruokonen and Kvist 2002; Yang et al. 2021). Among the ten conserved sequence elements, CSB1-like, CSB1, and LSP/HSP each had two variable sites, while the remaining seven were conserved across species (Fig. 5b), indicating the conserved functions of these regions (Delport et al. 2002).

### ﻿Mitogenomic phylogeny and taxonomic implications

**Figure 6.**
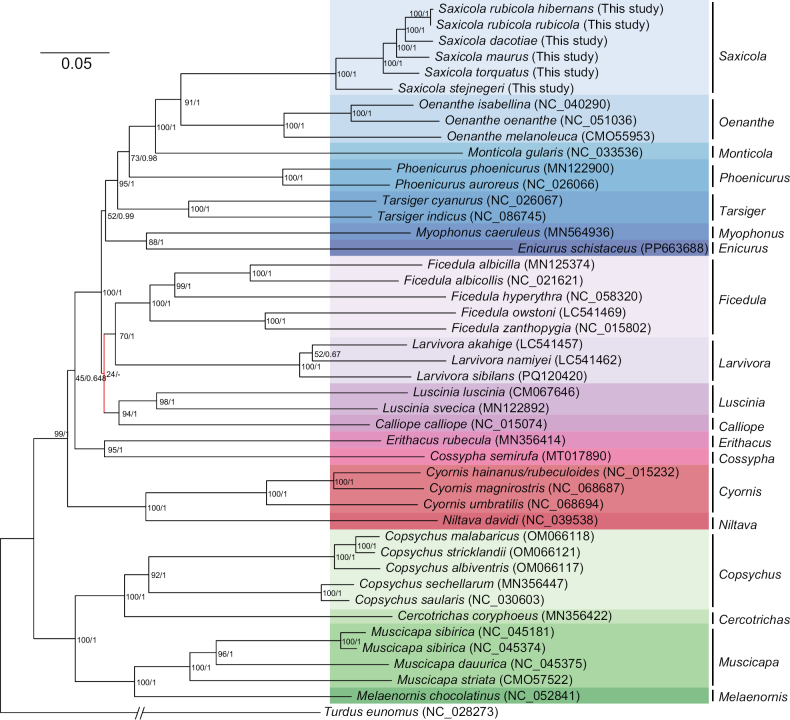
Phylogenetic tree based on 13 PCGs using ML and BI revealed the intra- and intergeneric phylogenetic relationships of *Saxicola* and supported the recognition of *S.stejnegeri* as a distinct species. The topological structure obtained from ML is displayed. Node support values are listed at nodes in the order BP/PP. Nodes highlighted in red indicate topological conflicts with the BI method (Suppl. material 8: fig. S2). Branch lengths of the outgroup are shortened for better visualization.

The family Muscicapidae, comprising 303–343 species, stood as one of the largest avian families, but the phylogenetic relationships within the family were still not fully resolved (Sangster et al. 2010; Zuccon and Ericson 2010; Zhao et al. 2023). Our phylogenetic analysis included all available mitogenomes of the Muscicapidae family (as of October 2024). Except for one weakly supported node, the ML and BI approaches showed the same topology (Fig. 6, Suppl. material 8: fig. S2). As expected, all species within the same genus clustered together as monophyletic, indicating the validity of their classification. *Saxicola* was most closely related to *Oenanthe*, forming sister genera with high node support (BP=93%, PP=1), which was consistent with previous studies (Zhao et al. 2023).

Within *Saxicola*, six taxa formed a nested phylogenetic structure: (((((*S.r.hibernans* + *S.r.rubicola*) + *S.dacotiae*) + *S.maurus*) + *S.torquatus*) + *S.stejnegeri*), all with the highest node support (BP = 100%, PP = 1) (Fig. 6). Notably, *S.stejnegeri*, as the sister group of all other sampled *Saxicola* taxa, did not cluster with *S.maurus*, aligning with prior *ND2*-based findings (Zink et al. 2009). *S.maurus* and *S.stejnegeri* were phenotypically nearly identical, differing slightly in plumage color, with *S.stejnegeri* being darker (Hellström and Waern 2023), but a study found no morphological or worn spring plumage distinctions, only notable differences in male song (Opaev et al. 2018). Their distribution areas were separated along the full length of the Yenisey/Angara rivers, where extensive hybridization occurred (Vaurie 1959; Hellström and Waern 2023), blurring the boundaries between the two species. Our mitogenome-based phylogeny with the highest node support, revealed an earlier split of *S.stejnegeri* from other Palearctic, African, and island stonechats before their divergence, supporting its status as an independent species rather than a subspecies of *S.maurus*.

### ﻿Divergence and biogeography in *Saxicola*

The *Saxicola* time tree, based on concatenated sequences of *ND2* and *Cytb*, covered 14 of the 15 phylogenetic species in this genus (missing *S.macrorhynchus*) (Gill et al. 2024), and the tree topology was consistent with both the mitogenome-based results and previous research (Illera et al. 2008; Woog et al. 2008; Zink et al. 2009; Zhao et al. 2023). Using a mutation rate of 0.0105 substitutions/site/million years for *Cytb* (Weir and Schluter 2008) and secondary calibration of two nodes as priors, the BI yielded an *ND2* mutation rate of 0.0148 substitutions/site/million years, which was slightly higher than that of *Cytb* (posterior rate = 0.0109 substitutions/site/million years). Divergence dating showed that stonechats diversified from the late Miocene to the early Pleistocene, forming all species-level lineages, similar to previous estimates (Illera et al. 2008; Zhao et al. 2023).

**Figure 7.**
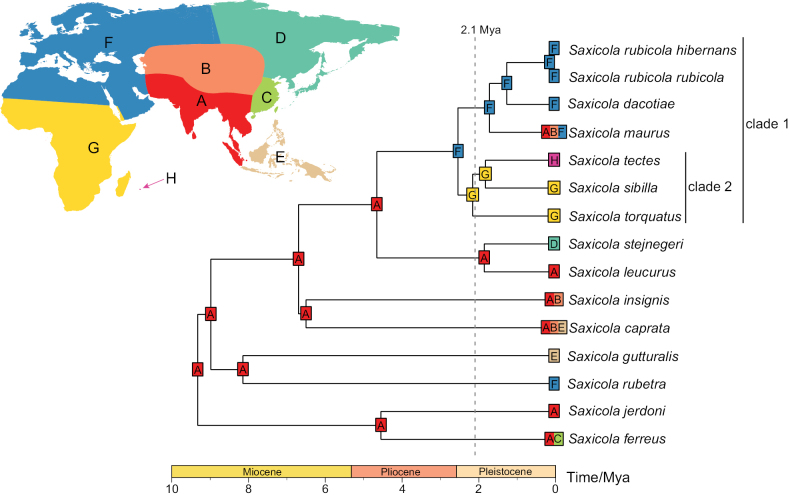
*Saxicola* biogeography analyzed with DEC + J model using *ND2 and Cytb* time-calibrated tree, supporting an origin in the Indian subcontinent and mainland Southeast Asia. The observed ranges of extant taxa are shown at the tips of the topology, with estimated ancestral ranges shown at the internal nodes. The vertical gray dashed lines indicate the timing of the uplift of Réunion Island. Letters on the map represent abbreviations for the eight biogeographic realms described in the Materials and methods section.

The evaluation of the six biogeographic models indicated that adding the founder event speciation parameter J (Matzke 2014) significantly improved the model fit and the DEC+J model showed the best fit for *Saxicola* (indicated by the highest weighted AIC) (Suppl. material 4: table S4). This model’s assumptions were the most realistic for *Saxicola* and were widely applied in biogeographic studies of other passerine birds (Oliveros et al. 2019; Batista et al. 2020; Schield et al. 2024). The biogeographic reconstruction (Fig. 7) suggested that *Saxicola* originated in the Indian subcontinent plus mainland Southeast Asia, supporting its Asian origin (Illera et al. 2008) with additional refinement. Multiple independent dispersal events and limited radiation occurred post-origin, with movements to Eastern Asia (*S.ferreus*), Western Palearctic (*S.rubetra*), Malay Archipelago (*S.gutturalis* and *S.caprata*), Central Asian arid (*S.caprata* and *S.insignis*), and Northeast Palearctic (*S.stejnegeri*). In contrast, a single dispersal to the Western Palearctic by the most recent common ancestor (MRCA) of the widely radiated clade 1 was suggested. Within clade 1, a reverse dispersal to the Indian subcontinent and an expansion into the Central Asian arid by the ancestors of *S.maurus* was proposed. This movement might have been driven by climatic fluctuations in Siberia caused by Pleistocene glacial events (Hewitt 1996, 2000), resulting in the adjacent distributions of *S.maurus* and *S.stejnegeri*, potentially facilitating hybridization between the two species (Hellström and Waern 2023). The MRCA of clade 2 dispersed from Western Palearctic to Africa, followed by a gradual colonization through Madagascar to Réunion Island. Notably, the divergence time between Madagascar stonechat *S.sibilla* and Réunion stonechat *S.tectes* was 1.86 million years ago (mya) (95% HPD = 1.28–2.47), which was close to the time (2.1 mya) of emergence of Réunion Island (Warren et al. 2003), suggesting that the uplift of the island might have facilitated the speciation of Réunion stonechat.

## ﻿Conclusions

In summary, we conducted the assembly and analysis of the mitogenomes of six *Saxicola* taxa, offering the first comprehensive insight into the mitogenomes of this genus. The six mitogenomes exhibited a conserved structure comprising 13 PCGs, 22 *tRNAs*, 2 *rRNAs*, and 1 *CR*, with characteristic AT bias and preferential A/C skewness. *ND3* was the fastest-evolving PCG, while *COX1* was the slowest. All *tRNAs* formed the classic cloverleaf structure except *trnS1* (AGY), and the *CR* had three domains with ten conserved elements. Additionally, our mitogenome-based phylogeny highlighted *S.stejnegeri* as a distinct lineage among the six *Saxicola* taxa, affirming its species separation from *S.maurus*. Furthermore, the divergence time estimates and biogeographic reconstruction based on *ND2* and *Cytb* suggested that *Saxicola* originated in the Indian subcontinent and mainland Southeast Asia in the late Miocene, and radiated in the early Pleistocene, resulting in a wide distribution across Eurasia and Africa. Sequencing mitogenomes of other *Saxicola* lineages is needed to provide a more comprehensive characterization and to increase the accuracy of phylogenetic and biogeographic reconstruction.

## ﻿Additional information

### Use of AI

No use of AI was reported.

### Author ORCIDs

Chuan Jiang https://orcid.org/0009-0002-5563-162X

Wenwen Zhu https://orcid.org/0009-0005-5433-4094

Shujia Wu https://orcid.org/0009-0006-0021-2777

Bo Li https://orcid.org/0000-0001-6824-4907

### Data availability

All data involved in this study has been uploaded to NCBI. The original sequencing data for *Saxicolastejnegeri* is under PRJNA1184580, and its annotated mitogenome is under PQ594835. The accession numbers for *Saxicoladacotiae*, *Saxicolamaurus*, *Saxicolarubicolahibernans*, *Saxicolarubicolarubicola*, and *Saxicolatorquatus* are BK068769, BK068770, BK068771, BK068772, and BK068773, respectively.

## Supplementary materials

Supplementary material 1 List of 45 species used for the mitogenomic phylogenetic analyses in this study Data type docx https://binary.pensoft.net/file/1393920This dataset is made available under the Open Database License (http://opendatacommons.org/licenses/odbl/1.0/). The Open Database License (ODbL) is a license agreement intended to allow users to freely share, modify, and use this Dataset while maintaining this same freedom for others, provided that the original source and author(s) are credited.Chuan Jiang, Wenwen Zhu, Shujia Wu, Xilong Zhao, Nassoro Mohamed, Bo Li

Supplementary material 2 The subset partitions, best models and sites used in mitogenomic phylogenetic analysis and divergence time estimation Data type docx Explanation note The values enclosed in parentheses following protein-coding genes indicate the positions of codons. https://binary.pensoft.net/file/1393921This dataset is made available under the Open Database License (http://opendatacommons.org/licenses/odbl/1.0/). The Open Database License (ODbL) is a license agreement intended to allow users to freely share, modify, and use this Dataset while maintaining this same freedom for others, provided that the original source and author(s) are credited.Chuan Jiang, Wenwen Zhu, Shujia Wu, Xilong Zhao, Nassoro Mohamed, Bo Li

Supplementary material 3 Gene list for divergence time estimation and biogeographical analysis of *Saxicola* Data type docx Explanation note ‘-’ indicates that the gene is not available. https://binary.pensoft.net/file/1393922This dataset is made available under the Open Database License (http://opendatacommons.org/licenses/odbl/1.0/). The Open Database License (ODbL) is a license agreement intended to allow users to freely share, modify, and use this Dataset while maintaining this same freedom for others, provided that the original source and author(s) are credited.Chuan Jiang, Wenwen Zhu, Shujia Wu, Xilong Zhao, Nassoro Mohamed, Bo Li

Supplementary material 4 Support for models of geographic range evolution fitted to the time-calibrated *Saxicola* tree Data type docx Explanation note D = rates of range expansion, E = range contraction, J = jump dispersal weight, AICc = Akaike information criterion score. https://binary.pensoft.net/file/1393923This dataset is made available under the Open Database License (http://opendatacommons.org/licenses/odbl/1.0/). The Open Database License (ODbL) is a license agreement intended to allow users to freely share, modify, and use this Dataset while maintaining this same freedom for others, provided that the original source and author(s) are credited.Chuan Jiang, Wenwen Zhu, Shujia Wu, Xilong Zhao, Nassoro Mohamed, Bo Li

Supplementary material 5 Sequencing depth and coverage map of six Saxicola mitogenomes demonstrated the structural integrity Data type eps https://binary.pensoft.net/file/1393924This dataset is made available under the Open Database License (http://opendatacommons.org/licenses/odbl/1.0/). The Open Database License (ODbL) is a license agreement intended to allow users to freely share, modify, and use this Dataset while maintaining this same freedom for others, provided that the original source and author(s) are credited.Chuan Jiang, Wenwen Zhu, Shujia Wu, Xilong Zhao, Nassoro Mohamed, Bo Li

Supplementary material 6 Mitogenomic structures of six *Saxicola* taxa Data type docx Explanation note The values separated by ‘/’ respectively represent the characteristics of *S.r.hibernans*, *S.r.rubicola*, *S.dacotiae*, *S.maurus*, *S.torquatus*, *S.stejnegeri.* https://binary.pensoft.net/file/1393925This dataset is made available under the Open Database License (http://opendatacommons.org/licenses/odbl/1.0/). The Open Database License (ODbL) is a license agreement intended to allow users to freely share, modify, and use this Dataset while maintaining this same freedom for others, provided that the original source and author(s) are credited.Chuan Jiang, Wenwen Zhu, Shujia Wu, Xilong Zhao, Nassoro Mohamed, Bo Li

Supplementary material 7 Compositions and biases of the mitogenomes of six *Saxicola* taxa in various datasets of mitogenomes Data type docx Explanation note The data corresponds to Fig. 1a. https://binary.pensoft.net/file/1393926This dataset is made available under the Open Database License (http://opendatacommons.org/licenses/odbl/1.0/). The Open Database License (ODbL) is a license agreement intended to allow users to freely share, modify, and use this Dataset while maintaining this same freedom for others, provided that the original source and author(s) are credited.Chuan Jiang, Wenwen Zhu, Shujia Wu, Xilong Zhao, Nassoro Mohamed, Bo Li

Supplementary material 8 Phylogenetic tree based on 13 PCGs using ML and BI revealed the intra- and intergeneric phylogenetic relationships of *Saxicola* and supported the recognition of *S.stejnegeri* as a distinct species Data type eps Explanation note The topological structure obtained from BI is displayed. Node support values are listed at nodes in the order BP/PP. https://binary.pensoft.net/file/1393927This dataset is made available under the Open Database License (http://opendatacommons.org/licenses/odbl/1.0/). The Open Database License (ODbL) is a license agreement intended to allow users to freely share, modify, and use this Dataset while maintaining this same freedom for others, provided that the original source and author(s) are credited.Chuan Jiang, Wenwen Zhu, Shujia Wu, Xilong Zhao, Nassoro Mohamed, Bo Li
