# Supplementary material for: ﻿Comparative mitogenomics, phylogeny, and biogeography of selected species of Saxicola (Aves, Passeriformes)
Source: Zookeys. 2025 Aug 13;1249:69–92. doi: 10.3897/zookeys.1249.152269 (PMC12368602; doi:10.3897/zookeys.1249.152269)
Supplement: Supplementary material 1 — List of 45 species used for the mitogenomic phylogenetic analyses in this study [file zookeys-1249-069_article-152269__-s001.docx]

**Table S1.** List of 45 species used for the mitogenomic phylogenetic analyses in this study.

| **Family** | **Species** | **GenBank No.** |
| --- | --- | --- |
| Muscicapidae | *Saxicola rubicola hibernans* | BK068771 (This study) |
|  | *Saxicola rubicola rubicola* | BK068772 (This study) |
|  | *Saxicola dacotiae* | BK068769 (This study) |
|  | *Saxicola maurus* | BK068770 (This study) |
|  | *Saxicola torquatus* | BK068773 (This study) |
|  | *Saxicola stejnegeri* | PQ594835 (This study) |
|  | *Calliope calliope* | NC_015074.1 |
|  | *Cercotrichas coryphoeus* | MN356422.1 |
|  | *Copsychus stricklandii* | OM066121.1 |
|  | *Copsychus albiventris* | OM066117.1 |
|  | *Copsychus malabaricus* | OM066118.1 |
|  | *Copsychus saularis* | NC_030603.1 |
|  | *Copsychus sechellarum* | MN356447.1 |
|  | *Cossypha semirufa* | MT017890.1 |
|  | *Cyornis hainanus/rubeculoides* | NC_015232.1 |
|  | *Cyornis magnirostris* | NC_068687.1 |
|  | *Cyornis umbratilis* | NC_068694 |
|  | *Enicurus schistaceus* | PP663688.1 |
|  | *Erithacus rubecula* | MN356414.1 |
|  | *Ficedula albicilla* | MN125374.1 |
|  | *Ficedula albicollis* | NC_021621.1 |
|  | *Ficedula hyperythra* | NC_058320.1 |
|  | *Ficedula owstoni* | LC541469.1 |
|  | *Ficedula zanthopygia* | NC_015802.1 |
|  | *Larvivora akahige* | LC541457.1 |
|  | *Larvivora namiyei* | LC541462.1 |
|  | *Larvivora sibilans* | PQ120420.1 |
|  | *Luscinia luscinia* | CM067646.1 |
|  | *Luscinia svecica* | MN122892.1 |
|  | *Melaenornis chocolatinus* | NC_052841.1 |
|  | *Monticola gularis* | NC_033536.1 |
|  | *Muscicapa dauurica* | NC_045375.1 |
|  | *Muscicapa sibirica* | NC_045374.1 |
|  | *Muscicapa sibirica* | NC_045181.1 |
|  | *Muscicapa striata* | CMO57522.1 |
|  | *Myophonus caeruleus* | MN564936.1 |
|  | *Niltava davidi* | NC_039538.1 |
|  | *Oenanthe isabellina* | NC_040290.1 |
|  | *Oenanthe melanoleuca* | CMO55953.1 |
|  | *Oenanthe oenanthe* | NC_051036.1 |
|  | *Phoenicurus auroreus* | NC_026066.1 |
|  | *Phoenicurus phoenicurus* | MN122900.1 |
|  | *Tarsiger cyanurus* | NC_026067.1 |
|  | *Tarsiger indicus* | NC_086745.1 |
| Turdidae | *Turdus eunomus* | NC_028273.1 |
