# Supplementary material for: ﻿Comparative mitogenomics, phylogeny, and biogeography of selected species of Saxicola (Aves, Passeriformes)
Source: Zookeys. 2025 Aug 13;1249:69–92. doi: 10.3897/zookeys.1249.152269 (PMC12368602; doi:10.3897/zookeys.1249.152269)
Supplement: Supplementary material 2 — The subset partitions, best models and sites used in mitogenomic phylogenetic analysis and divergence time estimation [file zookeys-1249-069_article-152269__-s002.docx]

**Table S2. The subset partitions, best models and sites used in mitogenomic phylogenetic analysis and divergence time estimation.** The values enclosed in parentheses following protein-coding genes indicate the positions of codons.

| **Analysis** | **Taxa number** | **Software** | **Subset** | **Best Model** | **Sites** | **Subset Partitions** |
| --- | --- | --- | --- | --- | --- | --- |
| Mitogenomic Phylogeny | 44 + Ourgroup | IQ-TREE | 1 | TVM+I+G | 1792 | *ATP6*(1), *ND2*(1), *ND4*(1), *ND4L*(1), *COX2*(3), *ATP8*(3), *Cytb*(3) |
|  |  |  | 2 | TIM+I+G | 1293 | *ATP6*(3), *ND2*(2), *ND3*(3), *ND5*(3) |
|  |  |  | 3 | TIM+G | 55 | *ATP8*(1), |
|  |  |  | 4 | GTR+I+G | 8218 | *ATP6*(2), *ATP8*(2), *ND1*(1), *ND1*(2), *ND1*(3), *ND2*(3), *ND3*(1), *ND3*(2), *ND4*(2), *ND4*(3), *ND4L*(2), *ND4L*(3), *ND5*(1), *ND5*(2), *ND6*(1) , *ND6*(2), *ND6*(3), *COX1*(1), *COX1*(2), *COX1*(3), *COX2*(1), *COX2*(2), *COX3*(1), *COX3*(2), *COX3*(3), *Cytb*(1), *Cytb*(2) |
|  |  |  | 1 | GTR+I+G | 11358 | *ATP6*(1), *ATP6*(2), *ATP6*(3), *ATP8*(1), *ATP8*(2), *ATP8*(3), *ND1*(1), *ND1*(2), *ND1*(3), *ND2*(1), *ND2*(2), *ND2*(3), *ND3*(1), *ND3*(2), *ND3*(3), *ND4*(1), *ND4*(2), *ND4*(3), *ND4L*(1), *ND4L*(2), *ND4L*(3), *ND5*(1), *ND5*(2), *ND5*(3), *ND6*(1), *ND6*(2), *ND6*(3), *COX1*(1), *COX1*(2), *COX1*(3), *COX2*(1), *COX2*(2), *COX2*(3), *COX3*(1), *COX3*(2), *COX3*(3), *Cytb*(1), *Cytb*(2), *Cytb*(3) |
| Divergence  and biogeography | 15 + Outgroup | BEAST | 1 | TRN+I+G | 381 | *Cytb*(1), |
|  |  |  | 2 | HKY+I | 381 | *Cytb*(2) |
|  |  |  | 3 | HKY+I+G | 381 | *Cytb*(3) |
|  |  |  | 4 | TRN+G | 347 | *ND2*(1) |
|  |  |  | 5 | TRN+I | 347 | *ND2*(2) |
|  |  |  | 6 | HKY+G | 347 | *ND2*(3) |
