# Supplementary material for: ﻿Comparative mitogenomics, phylogeny, and biogeography of selected species of Saxicola (Aves, Passeriformes)
Source: Zookeys. 2025 Aug 13;1249:69–92. doi: 10.3897/zookeys.1249.152269 (PMC12368602; doi:10.3897/zookeys.1249.152269)
Supplement: Supplementary material 3 — Gene list for divergence time estimation and biogeographical analysis of Saxicola [file zookeys-1249-069_article-152269__-s003.docx]

**Table S3. Gene list for divergence time estimation and biogeographical analysis of *Saxicola*.** ‘-’ indicates that the gene is not available.

| **Species** | ***ND2* (Sample Origin)** | ***Cytb* (Sample Origin)** |
| --- | --- | --- |
| *Saxicola rubicola hibernans* | This study (Kerry, Ireland) | This study (Kerry, Ireland) |
| *Saxicola rubicola rubicola* | This study (Lower Austria) | This study (Lower Austria) |
| *Saxicola dacotiae* | This study (Fuerteventura Island, Spain) | This study (Fuerteventura Island, Spain) |
| *Saxicola maurus* | This study (Germany) | This study (Germany) |
| *Saxicola torquatus* | This study (Nakuru, Kenya; Mount Meruregion, Tanzania) | This study (Nakuru, Kenya; Mount Meruregion, Tanzania) |
| *Saxicola stejnegeri* | This study (Beian, China) | This study (Beian, China) |
| *Saxicola sibilla* | EU190950 (Eastern Madagascar, Maromizaha) | EU190914 (Eastern Madagascar, Maromizaha) |
| *Saxicola leucurus* | KJ455634 (Punjab, India) | HM633375 (Punjab, India) |
| *Saxicola tectes* | EU190945 (Réunion Island) | EU421119 (Réunion Island) |
| *Saxicola caprata* | KJ455631 (Sulawesi, Indonesia) | KJ702870 (Western Ghats sky islands) |
| *Saxicola insignis* | - | EU421117 (Shuklaphanta, Nepal) |
| *Saxicola rubetra* | GU237123 (Sweden) | EU421115 (Iberian Peninsula, Spain) |
| *Saxicola gutturalis* | - | EU421125 (Timor Island) |
| *Saxicola ferreus* | KJ455632 (Uttar Pradesh, India) | KJ456455 (Uttar Pradesh, India) |
| *Saxicola jerdoni* | KJ455633 (Chiang Rai, Thailand) | EU427504 (Shuklaphanta, Nepal) |
| *Oenanthe oenanthe* | NC_051036 (Iceland) | NC_051036 (Iceland) |
