# Supplementary material for: ﻿Comparative mitogenomics, phylogeny, and biogeography of selected species of Saxicola (Aves, Passeriformes)
Source: Zookeys. 2025 Aug 13;1249:69–92. doi: 10.3897/zookeys.1249.152269 (PMC12368602; doi:10.3897/zookeys.1249.152269)
Supplement: Supplementary material 4 — Support for models of geographic range evolution fitted to the time-calibrated Saxicola tree [file zookeys-1249-069_article-152269__-s004.docx]

**Table S4.** Support for models of geographic range evolution fitted to the time-calibrated *Saxicola* tree. D = rates of range expansion, E = range contraction, J = jump dispersal weight, AICc = Akaike information criterion score.

| **Model** | **Numparams** | **LnL** | **D** | **E** | **J** | **AICc** | **AICc weight** |
| --- | --- | --- | --- | --- | --- | --- | --- |
| DEC | 2 | -44.47729 | 0.09228307 | 0.09501840 | 0.00000000 | 93.95458 | 0.0384740910 |
| DEC+J | 3 | -40.78489 | 0.03593471 | 0.02143486 | 0.13181493 | 89.75160 | 0.3146548262 |
| DIVALIKE | 2 | -43.01939 | 0.07338771 | 0.05721678 | 0.00000000 | 91.03878 | 0.1653207656 |
| DIVALIKE+J | 3 | -41.22760 | 0.04537765 | 0.02727864 | 0.09271803 | 90.63701 | 0.2021014986 |
| BAYAREALIKE | 2 | -48.28710 | 0.18557574 | 0.20918428 | 0.00000000 | 101.57420 | 0.0008522941 |
| BAYAREALIKE+j | 3 | -40.90660 | 0.03032409 | 0.05739800 | 0.11581911 | 89.99502 | 0.2785965245 |
