# Supplementary material for: ﻿Comparative mitogenomics, phylogeny, and biogeography of selected species of Saxicola (Aves, Passeriformes)
Source: Zookeys. 2025 Aug 13;1249:69–92. doi: 10.3897/zookeys.1249.152269 (PMC12368602; doi:10.3897/zookeys.1249.152269)
Supplement: Supplementary material 6 — Mitogenomic structures of six Saxicola taxa [file zookeys-1249-069_article-152269__-s006.docx]

**Table S5.** Mitogenomic structures of six *Saxicola* taxa. The values separated by ‘/’ respectively represent the characteristics of *S. r. hibernans*, *S. r. rubicola*, *S. dacotiae*, *S. maurus*, *S. torquatus*, *S. stejnegeri*.

| **Gene** | **Position** | | **Size (bp)** | **Orientatio** | **Intergenic Nucleotides(bp)** |
| --- | --- | --- | --- | --- | --- |
|  | **From** | **To** |  |  |  |
| *trnF* | 1/1/1/1/1/1 | 68/68/68/68/68/68/ | 68/68/68/68/68/68 | +/+/+/+/+/+ | 0/0/0/0/0/0 |
| *rrnS* | 68/68/68/68/68/68 | 1,049/1,049/1,049/1,049/1,050/1,051 | 982/982/982/982/983/984 | +/+/+/+/+/+ | -1/-1/-1/-1/-1/-1 |
| *trnV* | 1,049/1,049/1,049/1,049/1,050/1,051 | 1,118/1,118/1,118/1,118/1,119/1,120 | 70/70/70/70/70/70 | +/+/+/+/+/+ | -1/-1/-1/-1/-1/-1 |
| *rrnL* | 1,135/1,135/1,135/1,135/1,136/1,137 | 2,716/2,716/2,716/2,716/2,718/2,717 | 1582/1582/1582/1582/1583/1581 | +/+/+/+/+/+ | 16/16/16/16/16/16 |
| *trnL1*(UUR) | 2,718/2,718/2,718/2,718/2,720/2,719 | 2,792/2,792/2,792/2,792/2,794/2,793 | 75/75/75/75/75/75 | +/+/+/+/+/+ | 1/1/1/1/1/1/1 |
| *ND1* | 2,798/2,798/2,798/2,798/2,800/2,799 | 3,775/3,775/3,775/3,775/3,777/3,776 | 978/978/978/978/978/978 | +/+/+/+/+/+ | 5/5/5/5/5/5 |
| *trnI* | 3,780/3,780/3,780/3,780/3,782/3,781 | 3,851/3,851/3,851/3,851/3,853/3,852 | 72/72/72/72/72/72 | +/+/+/+/+/+ | 4/4/4/4/4/4 |
| *trnQ* | 3,857/3,857/3,857/3,857/3,859/3,858 | 3,927/3,927/3,927/3,927/3,929/3,928 | 71/71/71/71/71/71 | -/-/-/-/-/- | 5/5/5/5/5/5 |
| *trnM* | 3,927/3,927/3,927/3,927/3,929/3,928 | 3,995/3,995/3,995/3,995/3,997/3,996 | 69/69/69/69/69/69  /69  /69 | +/+/+/+/+/+ | -1/-1/-1/-1/-1/-1 |
| *ND2* | 3,996/3,996/3,996/3,996/3,998/3,997 | 5,036/5,036/5,036/5,036/5,038/5,037 | 1041/1041/1041/1041/1041/1041 | +/+/+/+/+/+ | 0/0/0/0/0/0 |
| *trnW* | 5,036/5,036/5,036/5,036/5,038/5,037 | 5,106/5,106/5,106/5,106/5,108/5,107 | 71/71/71/71/71/71 | +/+/+/+/+/+ | -1/-1/-1/-1/-1/-1 |
| *trnA* | 5,108/5,108/5,108/5,108/5,110/5,109 | 5,176/5,176/5,176/5,176/5,178/5,177 | 69/69/69/69/69/69 | -/-/-/-/-/- | 1/1/1/1/1/1 |
| *trnN* | 5,181/5,181/5,181/5,181/5,183/5,182 | 5,253/5,253/5,253/5,253/5,255/5,254 | 73/73/73/73/73/73 | -/-/-/-/-/- | 4/4/4/4/4/4 |
| *trnC* | 5,254/5,254/5,254/5,254/5,256/5,255 | 5,320/5,320/5,320/5,320/5,323/5,320 | 67/67/67/67/68/66 | -/-/-/-/-/- | 0/0/0/0/0/0 |
| *trnY* | 5,320/5,320/5,320/5,320/5,323/5,320 | 5,390/5,390/5,390/5,390/5,393/5,390 | 71/71/71/71/71/71 | -/-/-/-/-/- | -1/-1/-1/-1/-1/-1 |
| *COX1* | 5,392/5,392/5,392/5,392/5,395/5,392 | 6,942/6,942/6,942/6,942/6,945/6,942 | 1551/1551/1551/1551/1551/1551 | +/+/+/+/+/+ | 1/1/1/1/1/1 |
| *trnS2*(UCN) | 6,934/6,934/6,934/6,934/6,937/6,934 | 7,008/7,008/7,008/7,008/7,011/7,008 | 75/75/75/75/75/75 | -/-/-/-/-/- | -9/-9/-9/-9/-9/-9 |
| *trnD* | 7,013/7,013/7,013/4,013/7,016/7,013 | 7,081/7,081/7,081/7,081/7,084/7,081 | 69/69/69/69/69/69 | +/+/+/+/+/+ | 4/4/4/4/4 |
| *COX2* | 7,089/7,089/7,089/7,089/7,092/7,089 | 7,772/7,772/7,772/7,772/7,775/7,772 | 684/684/684/684/684/684 | +/+/+/+/+/+ | 7/7/7/7/7/7 |
| *trnK* | 7,774/7,774/7,774/7,774/7,777/7,774 | 7,842/7,842/7,842/7,842/7,845/7,842 | 69/69/69/69/69/69 | +/+/+/+/+/+ | 1/1/1/1/1/1 |
| *ATP8* | 7,844/7,844/7,844/7,844/7,847/7,844 | 8,011/8,011/8,011/8,011/8,014/8,011 | 168/168/168/168/168/168 | +/+/+/+/+/+ | 1/1/1/1/1/1 |
| *ATP6* | 8,002/8,002/8,002/8,002/8,005/8,002 | 8,685/8,685/8,685/8,685/8,688/8,685 | 684/684/684/684/684/684 | +/+/+/+/+/+ | -10/-10/-10/-10/-10/-10 |
| *COX3* | 8,691/8,691/8,691/8,691/8,694/8,691 | 9,474/9,474/9,474/9,474/9,477/9,474 | 784/784/784/784/784/784 | +/+/+/+/+/+ | 5/5/5/5/5/5 |
| *trnG* | 9,475/9,475/9,475/9,475/9,478/9,475 | 9,543/9,543/9,543/9,543/9,546/9,543 | 69/69/69/69/69/69 | +/+/+/+/+/+ | 1/1/1/1/1/1 |
| *ND3* | 9,544/9,544/9,544/9,544/9,547/9,544 | 9,894/9,894/9,894/9,894/9,897/9,894 | 351/351/351/351/351/351 | +/+/+/+/+/+ | 1/1/1/1/1/1 |
| *trnR* | 9,896/9,896/9,896/9,896/9,899/9,896 | 9,965/9,965/9,965/9,965/9,968/9,965 | 70/70/70/70/70/70 | +/+/+/+/+/+ | 1/1/1/1/1/1 |
| *ND4L* | 9,967/9,967/9,967/9,967/9,970/9,967 | 10,263/10,263/10,263/10,263/10,266/10,263 | 297/297/297/297/297/297 | +/+/+/+/+/+ | 1/1/1/1/1/1 |
| *ND4* | 10,257/10,257/10,257/10,257/10,260/10,257 | 11,634/11,634/11,634/11,634/11,637/11,634 | 1378/1378/1378/1378/1378/1378 | +/+/+/+/+/+ | -7/-7/-7/-7/-7/-7 |
| *trnH* | 11,635/11,635/11,635/11,635/11,638/11,635 | 11,705/11,705/11,705/11,705/11,708/11,705 | 71/71/71/71/71/71 | +/+/+/+/+/+ | 0/0/0/0/0/0 |
| *trnS1*(AGY) | 11,706/11,706/11,706/11,706/11,709/11,706 | 11,772/11,772/11,772/11,772/11,775/11,772 | 67/67/67/67/67/67 | +/+/+/+/+/+ | 0/0/0/0/0/0 |
| *trnL2*(CUN) | 11,772/11,772/11,772/11,772/11,775/11,772 | 11,842/11,842/11,842/11,842/11,845/11,842 | 71/71/71/71/71/71 | +/+/+/+/+/+ | -1/-1/-1/-1/-1/-1 |
| *ND5* | 11,843/11,843/11,843/11,843/11,846/11,843 | 13,660/13,660/13,660/13,660/13,663/13,660 | 1818/1818/1818/1818/1818/1818 | +/+/+/+/+/+ | 0/0/0/0/0/0 |
| *Cytb* | 13,669/13,669/13,669/13,669/13,672/13,669 | 14,811/14,811/14,811/14,811/14,814/14,811 | 1143/1143/1143/1143/1143/1143 | +/+/+/+/+/+ | 8/8/8/8/8/8 |
| *trnT* | 14,815/14,815/14,815/14,815/14,818/14,815 | 14,883/14,883/14,883/14,883/14,886/14,883 | 69/69/69/69/69/69 | +/+/+/+/+/+ | 3/3/3/3/3/3 |
| *trnP* | 14,890/14,890/14,890/14,890/14,893/14,890 | 14,959/14,959/14,959/14,959/14,962/14,959 | 70/70/70/70/70/70 | -/-/-/-/-/- | 6/6/6/6/6/6 |
| *ND6* | 14,969/14,969/14,969/14,969/14,972/14,969 | 15,487/15,487/15,487/15,487/15,490/15,487 | 519/519/519/519/519/519 | -/-/-/-/-/- | 9/9/9/9/9/9 |
| *trnE* | 15,489/15,489/15,489/15,489/15,492/15,489 | 15,560/15,560/15,560/15,560/15,563/15,560 | 72/72/72/72/72/72 | -/-/-/-/-/- | 1/1/1/1/1/1 |
| *CR* | 15,561/15,561/15,561/15,561/15,564/15,561 | 16,777/16,780/16,780/16,787/16,764/16,804 | 1217/1220/1220/1227/1201/1244 | +/+/+/+/+/+ | 0/0/0/0/0/0 |
