# Supplementary material for: ﻿Comparative mitogenomics, phylogeny, and biogeography of selected species of Saxicola (Aves, Passeriformes)
Source: Zookeys. 2025 Aug 13;1249:69–92. doi: 10.3897/zookeys.1249.152269 (PMC12368602; doi:10.3897/zookeys.1249.152269)
Supplement: Supplementary material 7 — Compositions and biases of the mitogenomes of six Saxicola taxa in various datasets of mitogenomes [file zookeys-1249-069_article-152269__-s007.docx]

**Table S6.** Compositions and biases of the mitogenomes of six *Saxicola* taxa in various datasets of mitogenomes. The data corresponds to Fig. 1a.

| **Compositions and biases** | **Species** | **Total genome** | **PCGs** | ***tRNAs*** | ***rRNAs*** | ***CR*** |
| --- | --- | --- | --- | --- | --- | --- |
| AT content | *Saxicola rubicola hibernans* | 53.26 | 52.20 | 57.02 | 53.32 | 56.29 |
|  | *Saxicola rubicola rubicola* | 53.30 | 52.28 | 57.02 | 53.32 | 56.15 |
|  | *Saxicola dacotiae* | 53.49 | 52.62 | 57.15 | 53.46 | 55.82 |
|  | *Saxicola maurus* | 53.32 | 52.54 | 57.34 | 53.23 | 55.75 |
|  | *Saxicola torquatus* | 53.16 | 52.14 | 57.44 | 53.34 | 56.12 |
|  | *Saxicola stejnegeri* | 53.03 | 52.03 | 56.73 | 53.14 | 55.63 |
| GC content | *Saxicola rubicola hibernans* | 46.74 | 47.80 | 42.92 | 46.68 | 43.71 |
|  | *Saxicola rubicola rubicola* | 46.70 | 47.72 | 42.98 | 46.68 | 43.85 |
|  | *Saxicola dacotiae* | 46.51 | 47.37 | 42.86 | 46.54 | 44.18 |
|  | *Saxicola maurus* | 46.69 | 47.46 | 42.66 | 46.77 | 44.25 |
|  | *Saxicola torquatus* | 46.84 | 47.86 | 42.56 | 46.66 | 43.88 |
|  | *Saxicola stejnegeri* | 46.97 | 47.97 | 43.28 | 46.86 | 44.37 |
| AT skew | *Saxicola rubicola hibernans* | 0.0846 | 0.0006 | 0.0413 | 0.2122 | -0.0774 |
|  | *Saxicola rubicola rubicola* | 0.0841 | -0.0004 | 0.0433 | 0.2122 | -0.0774 |
|  | *Saxicola dacotiae* | 0.0777 | -0.0080 | 0.0359 | 0.2021 | -0.0837 |
|  | *Saxicola maurus* | 0.0816 | -0.0001 | 0.0380 | 0.2066 | -0.0819 |
|  | *Saxicola torquatus* | 0.0926 | 0.0049 | 0.0406 | 0.2062 | -0.0683 |
|  | *Saxicola stejnegeri* | 0.0942 | 0.0158 | 0.0387 | 0.2125 | -0.0636 |
| GC skew | *Saxicola rubicola hibernans* | -0.3557 | -0.3667 | 0.0107 | -0.1285 | -0.3233 |
|  | *Saxicola rubicola rubicola* | -0.3558 | -0.3663 | 0.0097 | -0.1285 | -0.3271 |
|  | *Saxicola dacotiae* | -0.3509 | -0.3614 | 0.0162 | -0.1225 | -0.3135 |
|  | *Saxicola maurus* | -0.3546 | -0.3719 | 0.0146 | -0.1212 | -0.3370 |
|  | *Saxicola torquatus* | -0.3605 | -0.3727 | 0.0157 | -0.1225 | -0.3169 |
|  | *Saxicola stejnegeri* | -0.3627 | -0.3766 | 0.0160 | -0.1240 | -0.3442 |
